# KnowRU: Knowledge Reuse via Knowledge Distillation in Multi-Agent Reinforcement Learning

**DOI:** 10.3390/e23081043

**Published:** 2021-08-13

**Authors:** Zijian Gao, Kele Xu, Bo Ding, Huaimin Wang

**Affiliations:** College of Computer, National University of Defense Technology, Changsha 410000, China; gaozijian19@nudt.edu.cn (Z.G.); dingbo@aliyun.com (B.D.); whm_w@163.com (H.W.)

**Keywords:** multi-agent reinforcement learning, knowledge reuse, knowledge distillation

## Abstract

Recently, deep reinforcement learning (RL) algorithms have achieved significant progress in the multi-agent domain. However, training for increasingly complex tasks would be time-consuming and resource intensive. To alleviate this problem, efficient leveraging of historical experience is essential, which is under-explored in previous studies because most existing methods fail to achieve this goal in a continuously dynamic system owing to their complicated design. In this paper, we propose a method for knowledge reuse called “KnowRU”, which can be easily deployed in the majority of multi-agent reinforcement learning (MARL) algorithms without requiring complicated hand-coded design. We employ the knowledge distillation paradigm to transfer knowledge among agents to shorten the training phase for new tasks while improving the asymptotic performance of agents. To empirically demonstrate the robustness and effectiveness of KnowRU, we perform extensive experiments on state-of-the-art MARL algorithms in collaborative and competitive scenarios. The results show that KnowRU outperforms recently reported methods and not only successfully accelerates the training phase, but also improves the training performance, emphasizing the importance of the proposed knowledge reuse for MARL.

## 1. Introduction

Reinforcement learning (RL) has made great progress in solving complicated tasks, such as Atari games [1], board games [2], and video-game playing [1]. With the compelling performance of single-agent models, multi-agent RL (MARL) tasks, such as collaboration and competition among multiple agents, have piqued the interest of researchers in several fields [3,4], as the applications of MARL seems to be evident.

Current MARL algorithms are still highly task-specific and lack the ability to generalize to new environments. Moreover, for resource-limited embedded systems, training the MARL system from scratch would be extremely time-consuming and resource intensive because of the MARL system’s high complexity. Efficient transfer and use of knowledge between tasks can alleviate the aforementioned issues; sustainable efforts have been made in this field. One category of solutions employs the transfer learning paradigm to reuse the knowledge of historical experience, which can relieve the burden of training a new model with previous experience [5].

However, most existing transfer learning methods for multi-agents mainly depend on the hand-coded design, which requires knowledge from domain experts. For example, a method based on the Pepper algorithm [6] proposed by [7] is used to obtain strategies for opponents in adversarial scenarios and calculate policies against them. Then, agents in new scenarios evaluate the opponents’ strategy and reuse the knowledge by learning from the calculated policy. Similarly, a genetically programmed approach [8] utilizes strategies from previously trained networks to new tasks. A set of neural networks are trained to predict the value of each action and obtain a set of action strategies to build a multi-tiered architecture for agents to learn when to trigger which strategy in new tasks. However, these approaches mainly rely on hand-coded design, which is closely related to specific tasks, such as the mapping of state variables or modeling of opponents. Obviously, owing to the difficulties in professional and hand-coded design for tasks, existing methods that require sufficient domain knowledge are not universal enough and are difficult to deploy. It is desirable to reuse knowledge from previous experience using a method that can be widely used and easily deployed without considering how and what to transfer.

**Figure 1:**
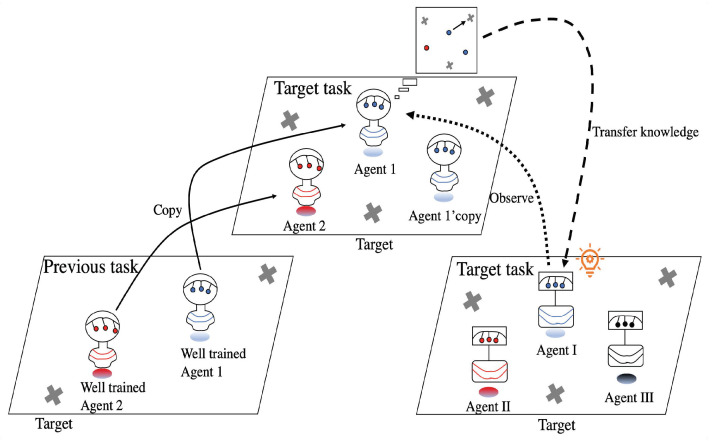
Overview of KnowRU. Agents in the target task mimic historical agents’ actions in the same situation, and then effectively reuse the knowledge in the previous task and allow further learning in a new environment.

In this paper, we propose a method for knowledge reuse called KnowRU, which can be easily deployed in MARL algorithms. KnowRU can shorten the training phase for new tasks while improving the asymptotic performance of agents. Our motivation is derived from knowledge distillation (KD) [9], which has been successfully applied in the field of computer vision. By leveraging the knowledge of well-trained agents in previous tasks as a historical experience, we employed the KD approach to transfer knowledge to agents for new tasks. Here, we suppose that the action taken by the agent in response to the environment is the simplest form of knowledge, and that action is determined by the network’s output. Therefore, mimicking the output is a potentially feasible way to reuse the knowledge of historical agents. During the training phase of new tasks, agents not only achieve higher rewards in the environment, but also mimic the outputs of previous agents. In this way, agents can learn from varying rewards and derive knowledge by mimicking historical agents. Knowledge from historical agents can be viewed as a fundamental consensus among different tasks owing to discrepancies in the tasks. Empirical experiments were conducted on different tasks and MARL algorithms to validate the effectiveness of KnowRU. Figure 1 illustrates an example in which agents must work together to reach the closest target as soon as possible. We retained the well-trained agents from the previous task; agents in the target task can observe how the well-trained agents would work in the same situation and transfer knowledge by mimicking the observed actions.

The contributions in this paper are as follows:

- We propose a task-independent KD framework for MARL, which focuses on not only accelerating the training phase, but also improving asymptotic performance for new tasks.
- Different strategies are explored to further improve the knowledge transfer performance.
- Extensive empirical experiments demonstrate the effectiveness of KnowRU in different task scenarios with different MARL algorithms.

## 2. Related Work

A multi-agent system (MAS) can be defined as stochastic games (Markov games, SG) [10], which extend from basic Markov decision processes (MDPs). Based on these theories, many excellent MARL algorithms have been proposed in recent years. The conventional approaches developed for basic MDPs, such as Q-Learning [11] and policy gradient [12], fail to train agents well in MAS because they have no head for considering environmental dynamics due to non-stationary policies of other agents. Instead, the MARL algorithms based on the actor–critic architecture [13], such as multi-agent deep deterministic policy gradient (MADDPG) [14], consider all agents’ information with a centralized critic. Furthermore, MAAC [15] learns a centralized critic with an attention mechanism to help agents focus on vital information.

However, because of the high sample complexity of conventional RL methods, training agents from scratch every time is difficult, especially in a continuous variational task, which would cost a considerable amount of time and computational resources. Transfer learning [16] is a practical approach to alleviate the problem via knowledge reuse. The vanilla MARL algorithms [17,18,19,20] focus only on designing some mechanism to improve the learning process of the current task. There are some methods aimed at mapping the relationships among tasks. For example, ref. [21] used the task relations to aggregate specific strategies in source tasks and generate an abstract policy that is used to help agents quickly adapt to new tasks. However, the indescribable relations among tasks are the difficulty of the abstracting policy. The use of evolutionary algorithms to transfer knowledge in an evolved multi-agent system is a feasible approach. Ref. [22] presented a neuro-evolutionary method that uses a neural network to codify agents’ policies, optimizes the network’s topology, and weights though interactions with respect to the environment. However, the method relies heavily on humans to define parameters and mapping between tasks. These above-mentioned transfer learning approaches differ from our method in the following ways. (1) They mainly focus on how and what to transfer between tasks. (2) The nifty artificial design for specific tasks based on sufficient domain knowledge is the key to their success. In this study, KnowRU only requires a policy model related to target tasks, without considering the model structure, relationships between tasks, or task-specific design. In comparison, KnowRU shows wider application prospects and can be aggregated into more MARL algorithms in a more convenient way.

KD is a type of knowledge transfer method, which was first proposed by [23] and has since become popular [9]. KD compresses the knowledge of large-scale complex models (*teachers*) into small and efficient models (*students*) to facilitate the deployment of models on devices with insufficient computing resources. The idea of KD inspired us to train the small student model with not only true labels but also soft targets provided by the well-performed large teacher model. Currently, the main KD methods can be divided into three categories: logits-based methods for learning the output layer, feature-based methods for learning the hidden layers, and relation-based methods for learning the relations between network layers. Some studies were also conducted on the application of KD in RL [24,25]. They mainly focus on making use of agent-level knowledge to tackle the problems in a single RL task with a KD paradigm. However, our primary focus was knowledge reusability in multi-agent tasks and to solve the problem of agents’ rapid adaptation to the dynamic changes of tasks in experiments.

## 3. Methodology

### 3.1. Preliminaries and Notations

MDPs [5] in MARL can be denoted as a tuple, $<S,U,T,R_{1\ldots n},\gamma >$, where *S* is the state space, *U* is the joint action space, *T* is the state transition function, $R_{i}$ is the reward function of agent *i*, γ is the discount factor, and *n* is the number of agents. The observation of agent *i* is in the current state $S_{i}$ is $O_{i}$; then, the agent takes action $A_{i}$ with policy $\pi_{\theta_{i}}$, and the environment produces the next state according to *T*. Agent *i* can obtain rewards $R_{i}$ from the environment according to state $S_{i}$ and action $A_{i}$. Agents simply aim to take proper actions that lead to maximal total return, $R=\sum_{t=0}^{T}\gamma^{t}R_{i}^{t}$, where *T* is the time horizon.

**Actor–Critic (AC):** To overcome high variance of policy gradient, the actor–critic [13] methods use a function as the critic to evaluate actions and replace the return term of the policy gradient with the value function. In RL, given the state and action, according to the Bellman equation, the critic function can be written as Equation (2), and the returns can be estimated so that the actor can be updated at every step. In this way, the models can be updated with less noise. The AC framework lays down a solid foundation for future multi-agent algorithms. Let π in Equation (2) be the agent’s policy.

(1) $$Q^{\pi }\left(s_{t},a_{t}\right)=E_{r_{t},s_{t+1}∼E}\left[r\left(s_{t},a_{t}\right)+\right.\left.\gamma E_{a_{t+1}∼\pi }\left[Q^{\pi }\left(s_{t+1},a_{t+1}\right)\right]\right].$$

**MADDPG:** MADDPG [14] is an extension of the deep deterministic policy gradient (DDPG) [26], which uses “target+online” networks and the experience replay to deal with the failure of AC in continuous action space. The critic in vanilla DDPG focuses only on the local information of the current agent, rather than the global view, resulting in a not-so-stable performance in multi-agent systems. Instead, MADDPG exploits global observations and actions for all agents with a centralized action-value function:

(2) $$Q_{i}^{\pi }\left(x,a_{1},\ldots ,a_{N}\right),$$

where $x=\left(o_{1},\ldots ,o_{N}\right)$ and *i* denotes the current agent. Let $\pi =\left\{\pi_{1},\ldots ,\pi_{N}\right\}$ be the set of all agent policies. If all actions taken by all agents are accessible, the learning processes conform to the Markov property. This is why MADDPG works well in multi-agent systems.

**MAAC:** MAAC [15] makes a significant contribution to the AC-based MARL algorithm with the attention mechanism. Every agent queries information from other agents’ observations and actions, and then estimates its value based on the information. The Q-value function, $Q_{i}^{\psi }$, considers the current agent *i*’s observation, action, and other agents’ contributions as follows:

(3) $$Q_{i}^{\psi }\left(o,a\right)=f_{i}\left(g_{i}\left(o_{i},a_{i}\right),x_{i}\right),$$

where $f_{i}$ is a multilayer perceptron (MLP), and $g_{i}$ is an MLP embedding function. The contribution from the key-value memory model is a weighted value of other agents:

(4) $$x_{i}=\sum_{j\neq i}\alpha_{j}v_{j}=\sum_{j\neq i}\alpha_{j}h\left(Vg_{j}\left(o_{j},a_{j}\right)\right),$$

where $v_{j}$ denotes a function of agent *j*’s embedding that is encoded with an embedding function, and then linearly transformed by shared matrix *V*; *h* is an element-wise nonlinearity; and $\alpha_{j}$ represents the attention weight.

In this study, we are in line with the AC methods and consider MADDPG and MAAC as our baselines.

### 3.2. KD

As mentioned earlier, because the teacher model provides more useful information for the student model, KD has achieved success in the field of computer vision. The soft probability output of trained teachers is key to distillation. Let $l_{t}$ be the input log of the final softmax layer of the teacher network, where $l_{t}=\left[l_{1},l_{2},\ldots ,l_{j}\right]$. The logits are converted into probabilities $q_{t}=\left[q_{1},q_{2},\ldots ,q_{j}\right]$ using the following softmax function: $q_{i}=\frac{e^{l_{i}}}{\sum_{j}e^{l_{j}}}$ where *i* represents the *i*-th neuron. To extract more information compared with true labels, [9] proposed softening the teacher probabilities with temperature *T*:

(5) $$q_{i}=\frac{exp(l_{i}/T)}{\sum_{j}exp\left(l_{j}/T\right)}.$$

In KD, such dark knowledge from the soft output of the teacher provides more information than that from the true labels. Based on the same image input *x*, the teacher and student networks produce probabilities $q_{t}\left(x\right)$ and $q_{s}\left(x\right)$ with Equation (5), respectively. The gap between $q_{t}\left(x\right)$ and $q_{s}\left(x\right)$ is usually penalized by the Kullback–Leibler (KL) divergence (Equation (7)) or cross-entropy loss:

(6) $$\begin{array}{c} L_{KD}=T^{2}KL\left(q_{s}\left(x\right),q_{t}\left(x\right)\right). \end{array}$$

(7) $$KL\left(P∥Q\right)=\sum P\left(x\right)\log\frac{P(x)}{Q(x)}$$

where*P(x) *and *Q(x)* are two probability distributions of the random variable *x*. Temperature *T* in Equation (6) also aims to soften the output of the teacher network. Then, through back propagation of $L_{KD}$, the student network could reuse knowledge from the teacher network. KD inspires us to believe that minimizing the gap between previous agents and current agents through the skill of distillation is the essence of knowledge reuse. We then draw upon the KD thought in our MARL research to reuse knowledge and verify the feasibility of KnowRU in Section 3.3.

### 3.3. Knowledge Reuse via Mimicking

Consider a real-world scenario in which our training task is constantly variational and the number of agents is also increasing. It is impractical to train agents from scratch whenever a task changes due to the high cost of time and resources. There are some similarities between tasks, and the knowledge learned in previous tasks can be regarded as historical experiences. Thus, knowledge reuse from historical experiences is important. However, we have argued that some existing works cannot reuse knowledge directly and effectively without exquisite design or expert-level experience. To solve such problems as rapid adaptation to dynamic changes in the number of agents, it is necessary to find an easier and more practical way of reusing knowledge. We aimed at designing a method that works well in such scenarios.

In this study, based on the concept of KD, we propose to reuse knowledge through mimicking to minimize the gap between tasks in MAS. We designed a task-independent knowledge reuse method that can be applied based on multiple MARL algorithms without a task-specific design. First, we used the policy models of the well-trained agents homogeneous with the current training agents in previous tasks related to the current task. Then, we paired every current agent and every homogeneous policy model. If the number of current agents is greater than the number of policy models, repeated pairings are allowed. During the training process, the same observations of the current agent were input into both the previous policy and current models. Based on the same input, the logits, which are the inputs of the last softmax layer, are used to measure the gap between tasks with a loss function. Simultaneously, current agents receive feedback from the environment through returns. It is worth mentioning that using logits and softmax is more of a direct and simple approach but using other characteristics of neural networks to measure the gap is also feasible.

**Figure 2:**
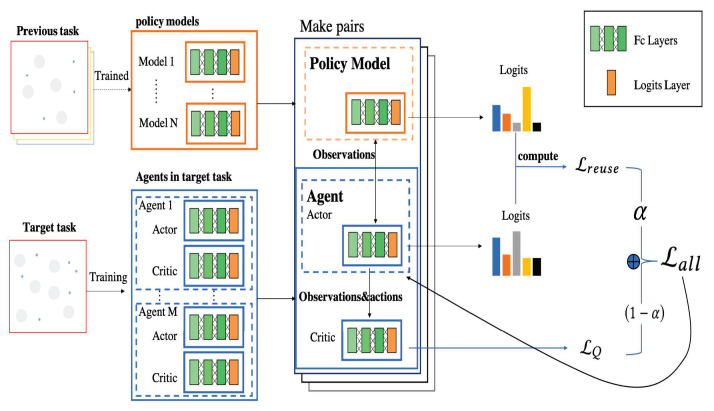
Workflow of KnowRU based on the actor–critic algorithm. It can be explained as three steps: 1: Match the policy models in the previous task with the agents in the target task. 2: Calculate the loss from the environment and the gap between the actor network and policy model. 3: While the agent receives feedback from the environment, it also mimics the actions of the policy model.

In this way, the agents of new tasks are trained by not only maximizing the total return from the environment but also by mimicking the output of previously well-trained policy models. Finally, considering the differences between tasks, determining how to reasonably combine the mimicking gap loss and returns from the environment is essential for training. Here, we used hyperparameter α to adjust the relationship between these two factors and finally obtain the total loss, $L_{all}$, for back propagation. Figure 2 illustrates the main components of KnowRU based on the AC framework that is widely used in MARL. KnowRU has been proven to be feasible in various experimental scenarios in Section 4.

**Mimicking.** In the field of computer vision, the teacher model used to transfer knowledge is a well-trained model that is trained on the same dataset as the current student model. Therefore, to extract dark knowledge, it is necessary to soften the teacher model’s output probability using Equation (5) and minimize the gap between them with cross-entropy (CE) loss or KL loss, using Equation (6). However, in the MARL, the source and target tasks might be different. The previous policy models may be overconfident or not authoritative in the new task. Therefore, it is not necessary to soften the probability of the policy model with hyperparameter *T*. Assume that $a_{p}$ and $a_{c}$ are the input logits of the final softmax layer in the previous policy model and current model, where $a_{p}=\left[a_{1},a_{2},\ldots ,a_{n}\right]$ and $a_{c}=\left[a_{1}^{\prime },a_{2}^{\prime },\ldots ,a_{n}^{\prime }\right]$, respectively. Here, we use the mean-squared error (MSE) loss as the $L_{reuse}$ loss function.

(8) $$L_{MSE}=\frac{1}{n}\sum_{i=1}^{n}{\left(a_{i}-a_{i}^{\prime }\right)}^{2}.$$

**Task’s guide and specialization.** We state that the previous policy model and current model usually work for different but similar tasks in MARL. The previous policy model cannot completely bootstrap the current learning; however, there is still some knowledge between similar tasks that can be described as a consensus. Some studies [27,28] showed that unprincipled reuse of knowledge may not help but hinder training. Therefore, it is vital to reuse the consensus in a reasonable manner. The training process is divided into two phases: phaseI **guide** and phaseII **specialization**. In phaseI, the previous policy model guides the current model to reuse knowledge. As the training progresses, the agents enter into the stage of **specialization,** stepwise, and the difference between tasks gradually increases. As shown in Figure 2, the training depends on two factors: $L_{reuse}$ and $L_{Q}$. We used hyperparameter α to adjust the weights of the two factors and achieve the transition between the two stages.

(9) $$L_{all}=\alpha L_{reuse}+\left(1-\alpha \right)L_{Q},$$

where α∈[0,1]. Here, $L_{reuse}:=L_{MSE}$, where $L_{Q}$ is defined as the Q-value obtained from the action-value function in the algorithms. By controlling α, we simulated the shift of focus from the guide phase to the specialisation phase in the learning process. In our experimental settings, α usually starts at around 0.5 and decreases linearly to 0.02. Some studies [29] showed that the low weight of knowledge reuse left behind can provide some noise for training to avoid task overfitting. It is worth mentioning that $L_{reuse}$ and $L_{Q}$ may not be of the same order of magnitude, and this may adversely affect RL. Our experiments demonstrated that scaling $L_{reuse}$ to an order of magnitude with $L_{Q}$ is a prudent solution to the problem. Algorithm 1 depicts the KnowRU algorithm based on the AC framework.
**Algorithm 1: **The training process based on AC framework.**Initialization:** Parameters $\theta_{A}$ and $\theta_{C}$ represent student’s actor and critic networks, respectively; parameter $\theta_{P}$ represents the previous policy model; and parameters α and β represent the weight of knowledge reuse and weight decay value, respectively. **Output:** Parameter $\theta_{A}$ of the actor networks and parameter $\theta_{C}$ of the critic networks for every agent: **for** episode = 1 to max-episodes:     **for** step *i* = 1 to max-steps-in-episode:       take action $a_{i}$: $a_{i}\;=\;\pi_{\theta_{A}}\left(s_{i}\right)+N_{i}$      get $r_{i}$ from environment, and observes new state $s_{i+1}$      store transition $\left(s_{i},a_{i},r_{i},s_{i+1}\right)$ into replay buffer    **end for**   randomly sample N transitions from replay buffer    **for** *j* = 1 to N by step k:       get $n_{j}$ samples by order       get $logits_{P}$ by $\pi_{\theta_{P}}\left(s_{i}\right)$      get $logits_{S}$ by $\pi_{\theta_{A}}\left(s_{i}\right)$       compute $L_{reuse}=\frac{1}{n_{j}}\sum_{i}\left(L_{function}\left(logits_{P},logits_{S}\right)\right)$      where $L_{function}$ := $L_{MSE}$      get $L_{Q}=\frac{1}{n_{j}}\sum_{i}\left(Q_{\theta_{C}}\left(s_{i},a_{i}\right)\right)$      optimize actor network by minimizing: $L_{all}=\alpha L_{reuse}+\left(1-\alpha \right)L_{Q}$       optimise critic network by minimizing: $L_{critic}=\frac{1}{n_{j}}\sum_{i}\left|\left(Q_{\theta_{C}}\left(s_{i},a_{i}\right)-r_{i}\right)\right|$   **end for**   **if** α>0.02:       α=α−β
**end for**

## 4. Experiments and Analysis

### 4.1. Experimental Setup

**Multi-agent particle environment.** We constructed three scenarios in the multi-agent particle environment (MPE) [14] to validate the performance of our method. The environment supports both cooperation and competition scenarios, and allows for the modification of existing scenarios. We tested KnowRU in both cooperation and competition scenarios based on the MADDPG and MAAC.

As mentioned earlier, knowledge reuse plays a vital role in continuous variational tasks. Therefore, we constructed a series of environments to simulate the variations in such tasks by changing the number of agents and landmarks. In our experiments, KnowRU had a positive impact on accelerating training and improving performance, primarily by comparing the results of basic algorithms and KnowRU. All the contrasts were based on the same experimental conditions. The experiments consisted of three scenarios involving cooperation and competition: simple_spread, simple_adversary, and cooperative_treasure_collection.

**Figure 3:**
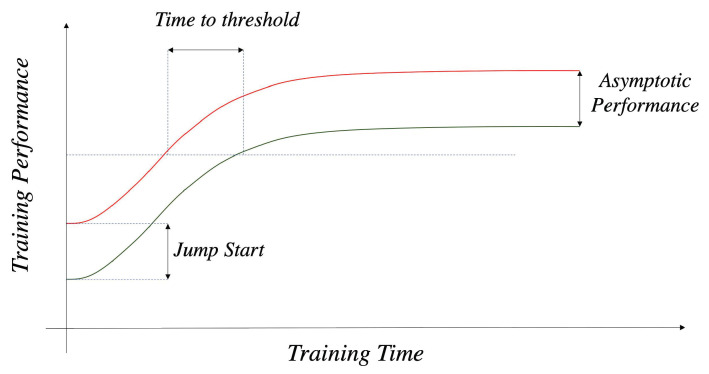
Transfer performance metrics.

**Performance Metrics.** There is also a common standard to measure the success of knowledge reuse in our experiments, summarized by [16] and resumed by [5], as illustrated in Figure 3. The three main indicators are as follows: (1) **jump start**—measuring performance improvement at the beginning of training; (2) **time to threshold**—for tasks which may yield a significant result at some point, the learning time required to reach the level is meaningful; and (3) **asymptotic performance**—in complex tasks, agents may fail to achieve optimal performance and instead achieve a suboptimal one. Knowledge reusing may help agents in achieving higher performance, and the before-and-after performance gap is called asymptotic performance.

### 4.2. Simple _Spread Scenario

A simple _spread is a predefined typical cooperative scenario of an MPE. In this environment, agents must cooperate to reach a set of landmarks (targets) without communication. The targets are not different, and agents must collect the shared rewards by arriving at all targets as quickly as possible. Meanwhile, agents are penalized when they collide with one another.

**Figure 4:**
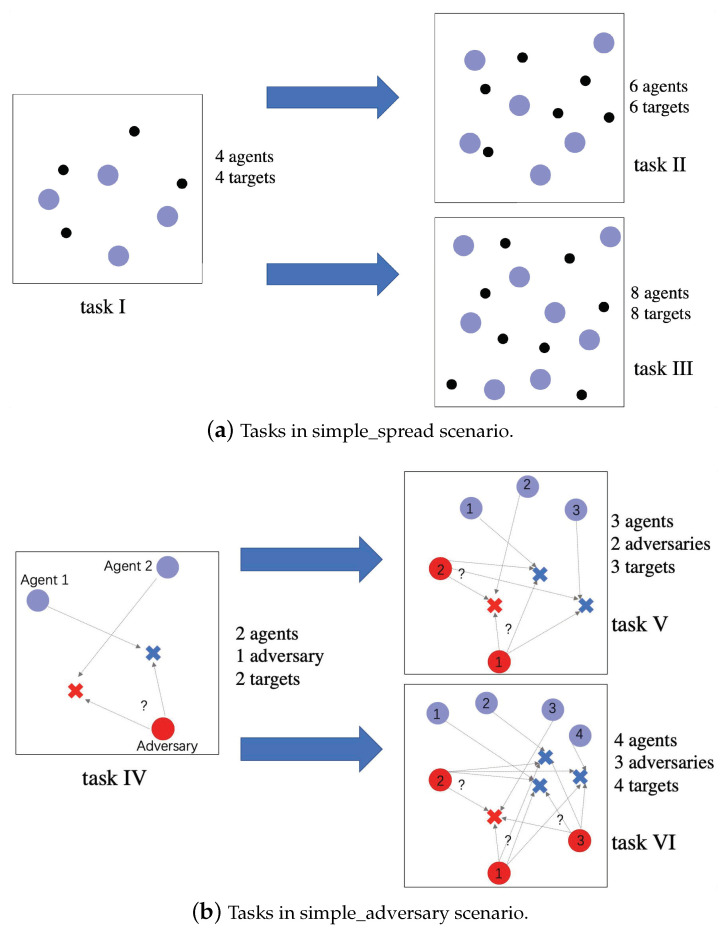
The tasks in Simple_spread & Simple_adversary.

In our experimental settings, we took the task containing four agents and four targets as the previous policy models’ training scenario, called Task I. In a real-world scenario, the number of agents and targets usually changes with the continued variation of the training mission. For this reason, we designed two scenarios to serve as current agents’ training scenarios. Task II was made up of six agents and six targets, while Task III was made up of eight agents and eight targets, as illustrated in Figure 4a. In simple_spread, we used MADDPG as the baseline algorithm and implemented KnowRU based on it. In addition, we specially added a control group that initialized the current agent with the previous policy model, which is called “MADDPG with initialization”, to verify the infeasibility of training directly based on the policy model.

### 4.3. Simple _Adversary Scenario

A simple _adversary is a predefined typical competitive MPE scenario. In this scenario, *n* agents must work together to reach one target landmark from the total *n* landmarks (targets) without communicating. The agents must maximize the shared rewards by minimizing the distance between the right target and the nearest agent. Meanwhile, the adversaries also want to reach the target without knowing which one is right, and the agents are also penalized by the adversary’s distance from the target. Because agents know the correct target point, the agents have an advantage at the beginning. As the training progressed, adversaries learned how to distinguish the correct target and achieve a balance of power.

In our experimental settings, we took the task that contained two agents, one adversary, and two targets as the previous policy models’ training scenario, called Task IV. We designed two scenarios as the current agents’ training scenarios. Task V was made up of three agents, two adversary and three targets, while Task VI was made up of three agents, two adversary and three targets as illustrated in Figure 4b. For this scenario, we only implemented KnowRU with adversaries to help them distinguish the correct target at the beginning.

**Figure 5:**
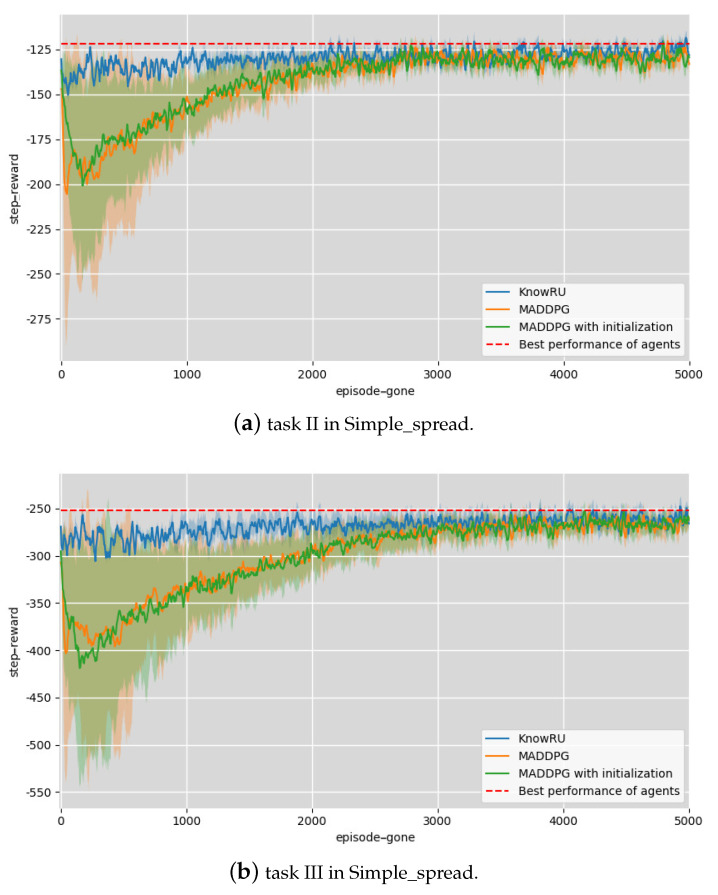
Knowledge reusing in Simple_spread.

**Figure 6:**
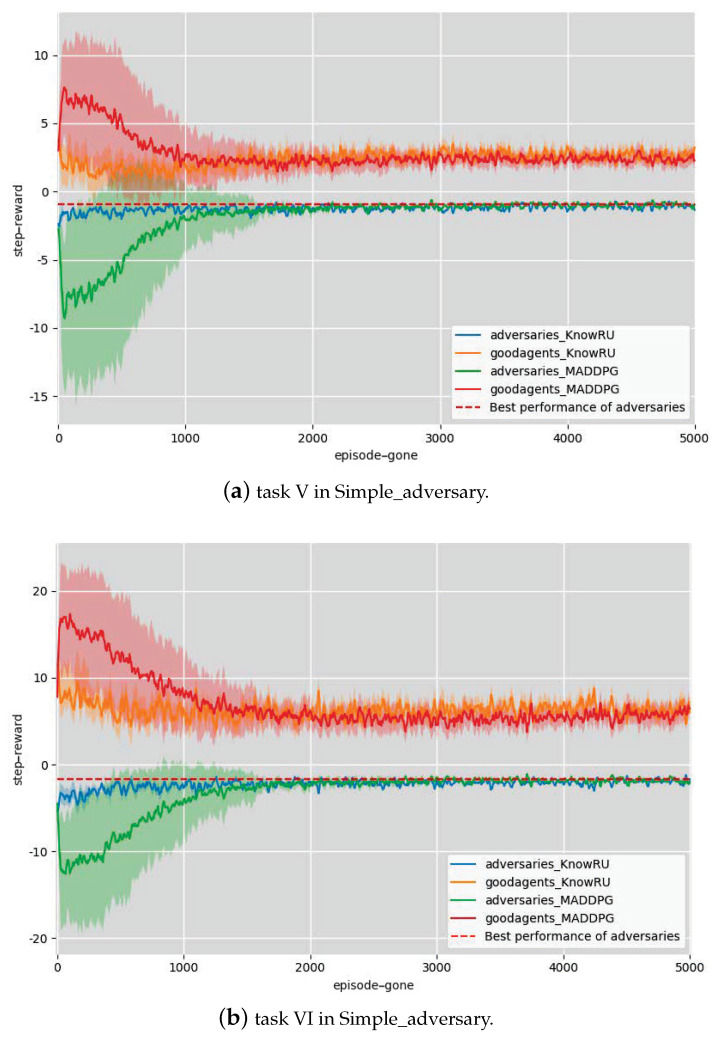
Knowledge reusing in Simple_adversary.

In the first two scenarios, we took the average step reward from the environments in each episode to measure the effect of training. The higher the reward, the better was the reward. The actor and critic networks in the experiments were all randomly parameterized by a four-layer fully connected MLP with 64 units per layer. The architecture of MLP is not strictly limited, only that the output layers of historical models and current models (that is, the neural layer’s output that determines the action) have the same action dimensions. We set 5000 episodes to ensure convergence, every episode consisted of 25 steps, and the networks were updated every four episodes. The hyperparameters α and *i* are set to 0.5 and 0.02, respectively. All the results and 95% confidence intervals (95% CI) are illustrated in Figure 5 and Figure 6. The red lines represent the best performances in these scenarios.

**Discussion.** From Figure 5 and Figure 6, it is obvious that the performance of KnowRU in all experiments was greatly improved at the beginning of training, and the episodes required for training convergence were also greatly reduced, satisfying two of the three indicators, and demonstrating that KnowRU successfully reuses knowledge. It is worth mentioning that when we trained the initialized agents in Tasks II and III, compared to MADDPG, the results did not clearly improve. This was caused by the characteristics of the models, which usually only work well in the scenarios where they are trained. When the task changes, the former models are no longer applicable because of over-fitting. Meanwhile, MADDPG showed considerable fluctuations in the training process without prior knowledge, particularly in the early stages of training, and performance decreased significantly. As for KnowRU, the convergence results were almost obtained at the beginning, achieving the goals of reusing knowledge in new tasks. Compared to MADDPG, KnowRU showed excellent performance and small fluctuations during training. We believe that the reason KnowRU works is that it effectively narrows the solution space by providing more prior knowledge, thereby narrowing the space for exploration. We also found that during the training process, the fluctuation of the loss function value caused an unstable performance of the agents. When we used KnowRU to train the agents, the oscillating amplitude of the loss value for backpropagation was apparently smaller, and $L_{reuse}$ first decreased and then increased, demonstrating the effectiveness of KnowRU’s two-phrase design. KnowRU does not appear to learn beyond its initial performance because agents achieved the best performance in the scenarios.

### 4.4. Cooperative _Treasure _Collection Scenario

Cooperative_treasure_collection is a more complex competitive scenario constructed by [15] based on the framework of the MPE. In this scenario, there are two types of agents: *X* treasure collectors and *Y* treasure banks. There are also *X* treasures that correspond to the color of the bank. The collector’s role is to collect the treasures of any color and transport them to the bank of the corresponding color. The treasures are reborn after being collected, and the banks simply gather as many treasures as possible from collectors. When the treasures are collected by the collectors, the collectors will share a global reward.

At the same time, while the treasure is transported into the bank, all agents receive a global reward. However, collectors are penalized if they collide with one another. To conclude, the collectors must learn how to collect treasures cooperatively and deposit them into the correct color bank as quickly as possible without colliding with other agents. Banks need to cooperate with collectors to place treasures.

**Figure 7:**
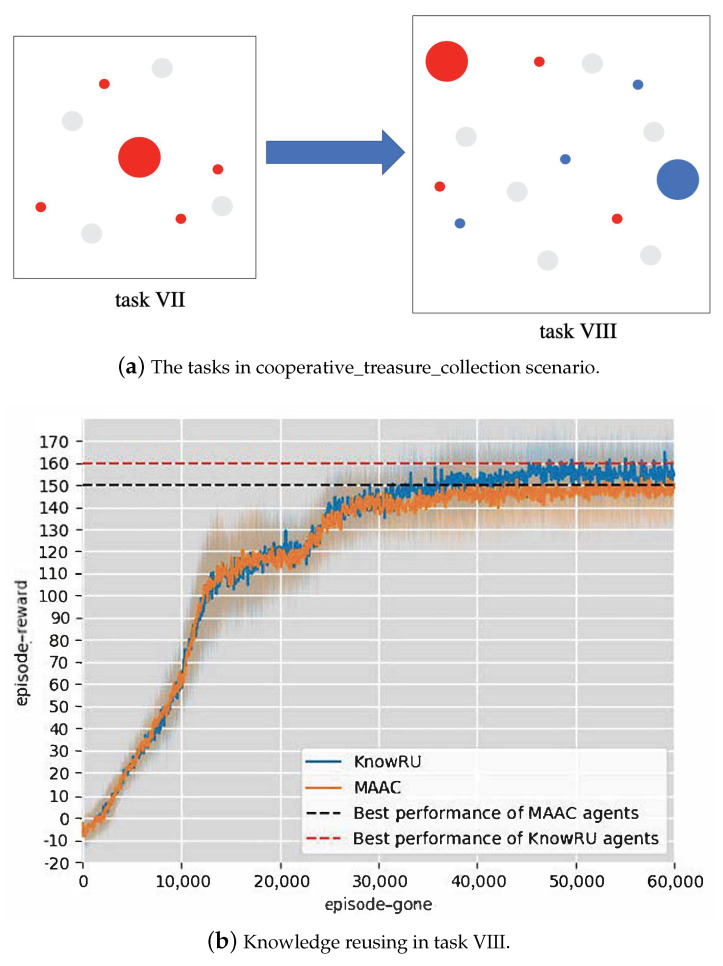
The tasks and results in Cooperative_treasure_collection.

In our experimental settings, we took the task which contained four collectors, one bank, and four treasures as the teacher models’ training scenario, called Task VII. We designed a scenario which consists of six collectors, two banks, and six treasures as student model training scenarios, called Task VIII, as shown in Figure 7a. To test the universality, we used MAAC as the basic algorithm, which can learn these tasks well using the attention mechanism and implement KnowRU based on it. The actor networks in our experiments were randomly parameterized by a four-layer fully connected MLP with 64 units per layer, and critic networks were parameterized by the attention mechanism. We set 60,000 episodes to ensure convergence; each episode consisted of 100 steps, and the networks were updated four times every episode. The hyperparameters α and *i* were set to 0.5 and 0.02, respectively. We took the average episode reward of all agents from the environment to measure the effect of training. The higher the reward, the better. The results and 95% confidence intervals (95% CI) are illustrated in Figure 7b.

**Discussion.** As shown in Figure 7b, we completed the time to threshold and asymptotic performance goals. MAAC first obtained and stabilized a reward value of 150 at approximately 37,000 episodes. However, KnowRU first achieved approximately 29,000 episodes and advanced this time by approximately 21.6% compared to MAAC. In comparison to the other two scenarios, we can draw different conclusions and conjectures from this scenario. Even though the two tasks in this scenario are not closely related, KnowRU also successfully helps agents in avoiding the local optima and achieving higher performance. The result proves our conjecture that KnowRU can help and guide the training of agents by providing more useful information.

### 4.5. Component Analysis and Discussion

**Figure 8:**
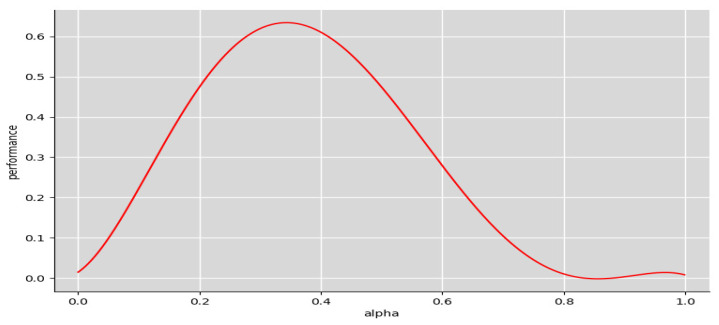
The impact of alpha on the moment when the best performance is achieved.

**Alpha.** We have declared that annealing of the alpha parameter blends the guide phase and specialization phase. We intended to explore the impact of alpha on performance using Equations (10) and (11).

(10) $$T=\left\{T_{\alpha 1},T_{\alpha 2},T_{\alpha 3}\cdots T_{\alpha n}\right\}$$

(11) $${\mathrm{Performance}}_{\alpha }=\ln\left(\frac{T_{\max}}{T_{\alpha }}\right)$$

where $T_{\alpha }$ represents the moment when the best performance is achieved with α, *T* is the set of $T_{\alpha }$, $T_{\max}$ is the maximum value of the set T, and performance ${}_{\alpha }$ denotes the performance of α. We tested the performance in Task III of the simple_spread scenario, and the results are shown in Figure 8.

When alpha is greater than 0.1, our method clearly outperforms the baseline. Here, we explored the best settings for alpha. When the alpha value is approximately 0.3∼0.5, the training performance is optimal, which is in line with our expectation that the training process is first guided and then specialized. We also found similar patterns in the other tasks.

**Loss Function.** There are three viable loss functions for $L_{reuse}$ tested: MSE loss, CE loss, and KL loss. The logits can be converted into probabilities with a softmax function, which can then be used in CE or KL. We found that using different loss functions had no discernible effect on the experimental results.

**Discussion.** We tried different combinations of the components. The results did not show a significant difference, and they all demonstrated that KnowRU successfully helped agents in quickly adapting to the environment, proving the effectiveness and robustness of our method.

## 5. Conclusions

The transferring of knowledge from historical experiences is of great practical importance in the MARL fields; however, it is notoriously unstable and difficult. In this study, we explored a novel knowledge transfer approach for MARL and addressed its accompanying unique challenges, leveraging the KD paradigm. To empirically demonstrate the robustness and effectiveness of KnowRU, we performed extensive experiments on state-of-the-art MARL algorithms in collaborative and competitive scenarios. The results demonstrate the effectiveness and robustness of KnowRU under different experimental settings.

## Data Availability

The source code of KnowRU is published on: https://github.com/KnowRU-MARL/KnowRU.
